# Evaluating the Paucity of Female Surgeons in Plastic Surgery: A Review of the Systemic Barriers to Entry and Success

**DOI:** 10.7759/cureus.75529

**Published:** 2024-12-11

**Authors:** Rijul S Maini, Savannah L Jelneck, William B Zimmerman

**Affiliations:** 1 Department of Osteopathic Medicine, Michigan State University College of Osteopathic Medicine, East Lansing, USA

**Keywords:** american society of plastic surgeons, female plastic surgeons, gender disparity, gender disparity in plastic surgery, plastic and reconstructive surgery, plastic surgery, plastic surgery residency, plastic surgery training, women in plastic surgery

## Abstract

The percentage of practicing female plastic surgeons in the United States is notably low. This narrative review sought to identify prominent barriers affecting women’s entry and success in plastic surgery. A literature search was conducted using the National Library of Medicine from 2013 to 2023, using MeSH terms of gender disparity and plastic surgery. Included publications were peer-reviewed articles and systematic reviews evaluating gender disparity in plastic surgery, examining experiences, challenges, or opportunities for female plastic surgeons. Excluded publications did not include data gathered from the United States, included other minorities, or did not focus on plastic surgery. Overall, 191 papers were identified, with 14 papers being selected for this review. Early barriers identified before residency training include the lack of female mentors and the underrepresentation of female presenters at national plastic surgery conferences, with females comprising only 29% of presenters and 16% of abstract senior authors at national plastic surgery conferences between 2014 and 2015. During residency training, the most prominent barrier is pregnancy, with 73% of women delaying childbearing during residency and only 39% of men reporting the same. A second barrier during training includes gender disparity in the number of research publications, with females publishing 8.89 ± 0.97 publications during residency and males publishing 12.46 ± 1.08 publications (p = 0.0394). After residency training, evidence of barriers to career advancement includes poor representation of women in higher academic positions, such as program chairs and directors, with female representation of 9.2% and 13.1%, respectively, and gender disparity in industry payments. Systemic barriers before, during, and after plastic surgery residency training seem to influence the representation of women in plastic surgery at all levels. These barriers should be addressed to increase the number of practicing female plastic surgeons and diversify the field.

## Introduction and background

The first female plastic surgeon in the United States was Dr. Alma Dea Morani, who applied for plastic surgery training six different times over six years, until she was accepted to a position with shadowing-only privileges. She overcame obstacles with remarkable determination and perseverance, becoming a member of the American Society of Plastic Surgeons (ASPS) in 1948 and building a successful career in plastic surgery. In 1992, the Women Plastic Surgeons Forum within ASPS was established, which fostered mentorship among emerging female leaders and allowed women to take on leadership roles within national plastic surgery organizations. These women have become role models for subsequent generations of women in plastic surgery [1].

Data from the Association of American Medical Colleges (AAMC) shows that, as of 2023, women make up 54.6% of total enrolled medical students in the United States [2]. In 2023, the proportion of female and male applicants applying to integrated plastic surgery residency was similar, with 196 (48%) female applicants and 210 (52%) male applicants [3]. However, the gender disparity in surgical specialties persists, despite medical school classes being composed of a female majority. The plastic surgery specialty is consistent with this trend. Of over 7,000 practicing plastic surgeons in the United States in 2021, less than 18% were female [4].

We hypothesize that barriers exist for women at every stage of their plastic surgery careers, contributing to the gender disparity seen among practicing plastic surgeons. This narrative review seeks to identify barriers to entry and success before, during, and after plastic surgery residency training for females, compared to males.

## Review

Methods 

Research Design 

**Figure 1:**
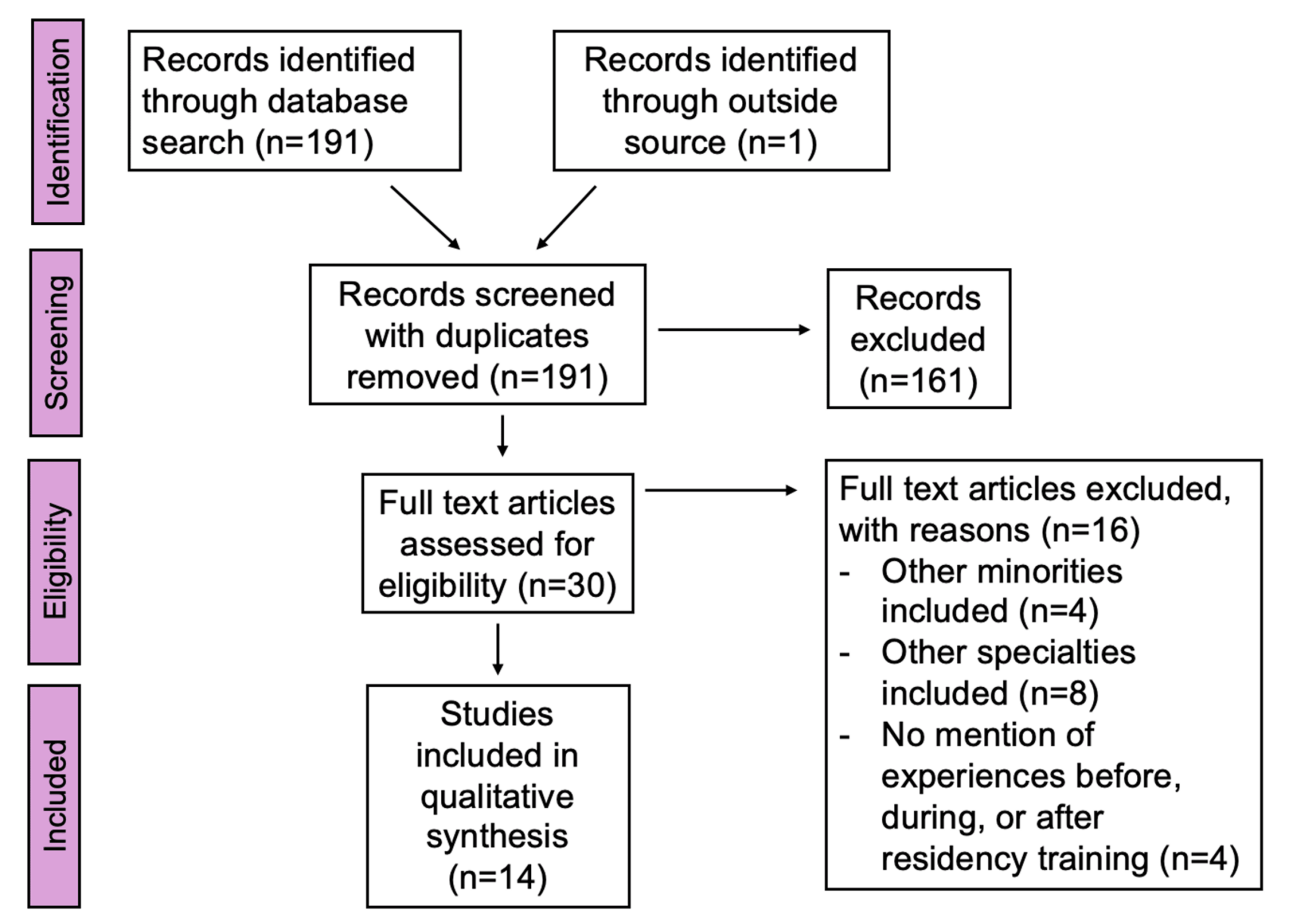
PRISMA flowchart PRISMA, Preferred Reporting Items for Systematic Review and Meta-Analyses

There are no standard guidelines for conducting a narrative review. Our review followed guidelines by the Preferred Reporting Items for Systematic Reviews and Meta-Analyses (PRISMA) criteria [5]. There is no protocol for this review, and this review is not registered. The PRISMA flow chart is shown in Figure 1.

Search Methods and Strategy

The National Library of Medicine Database was used to identify articles published between 2013 and 2023, consisting of peer-reviewed articles and systematic reviews evaluating gender disparity in plastic surgery, examining experiences, challenges, or opportunities for female plastic surgeons. The following terms were used without any limits: “gender disparity” and “plastic surgery.” The search used the English language and keywords combined with the Boolean logical operators “AND” to identify articles published between 2013 and 2023. The following terms were used without any limits: “gender disparity” and “plastic surgery.” Studies were excluded for the following reasons: no mention of experiences before, during, or after residency training; reporting women and other minority groups, such as races underrepresented in medicine (i.e., African American group, Hispanic group, Native American group), as one entity; or including non-plastic reconstructive surgery specialties (i.e., facial plastic surgery, otolaryngology, oculoplastic surgery, etc.). Studies using data from outside the United States were also excluded.

Data Extraction and Synthesis

Two reviewers (RM and SJ) independently evaluated all 191 titles, abstracts, and full texts to adjudicate whether each study met the inclusion criteria of the systematic review. Data were extracted from each study using a standardized data extraction form in Microsoft Excel 2016 (Microsoft® Corp., Redmond, WA, USA). We required that data extracted from the articles support systemic barriers in at least one of the three categories: before, during, or after plastic surgery residency training. Articles that did not include relevant information about these barriers were excluded from the analysis. The primary objective was to identify barriers women face in plastic surgery before, during, and after residency training. Disagreements between reviewers were resolved by consensus or by a third independent reviewer. Data were extracted from each paper and consolidated into an Excel document, with categories as follows: study type, timeline (before, during, or after training), barriers mentioned, and statistical data. After data extraction, narrative synthesis was performed to summarize the common themes and statistics found across studies.

Results

Overall, 14 studies were included for analysis, which included three survey studies (860 total respondents), five retrospective studies (across 99 plastic surgery programs, 153 institutions, and 1,518 plastic surgeons), four cross-sectional studies (analyzing between 316 and 818 academic plastic surgeons), one systematic review (analyzing 55 articles), and one peer-reviewed journal article.

The most prominent barriers found before residency training include a lack of female mentorship and unequal female representation of presenters at plastic surgery conferences. Barriers during plastic surgery residency include pregnancy and gender disparity in the number of publications. Evidence of barriers after residency training includes gender discrepancy in program chair and director positions and industry payments.

Barriers Identified Before Plastic Surgery Training

Early barriers include a lack of female role models demonstrating a work-life balance and an underrepresentation of female presenters at national plastic surgery conferences. In a survey using self-esteem and self-efficacy as proxies for confidence, 74% of female plastic surgeon respondents felt mentorship helped advance their careers and increase their professional confidence. Of the respondents with mentors (71%), less than half (42%) indicated their mentors were female. Having female mentors was associated with statistically significantly higher scores on the self-esteem survey (p = 0.032) [6].

An additional barrier identified before residency training was unequal representation at plastic surgery research conferences, where many presenters are in the early stages of their medical careers. Out of 1,171 abstracts presented at national plastic surgery conferences between 2014 and 2015, females comprised only 29% of presenters and 16% of abstract senior authors [7].

Barriers Identified During Plastic Surgery Training

**Figure 2:**
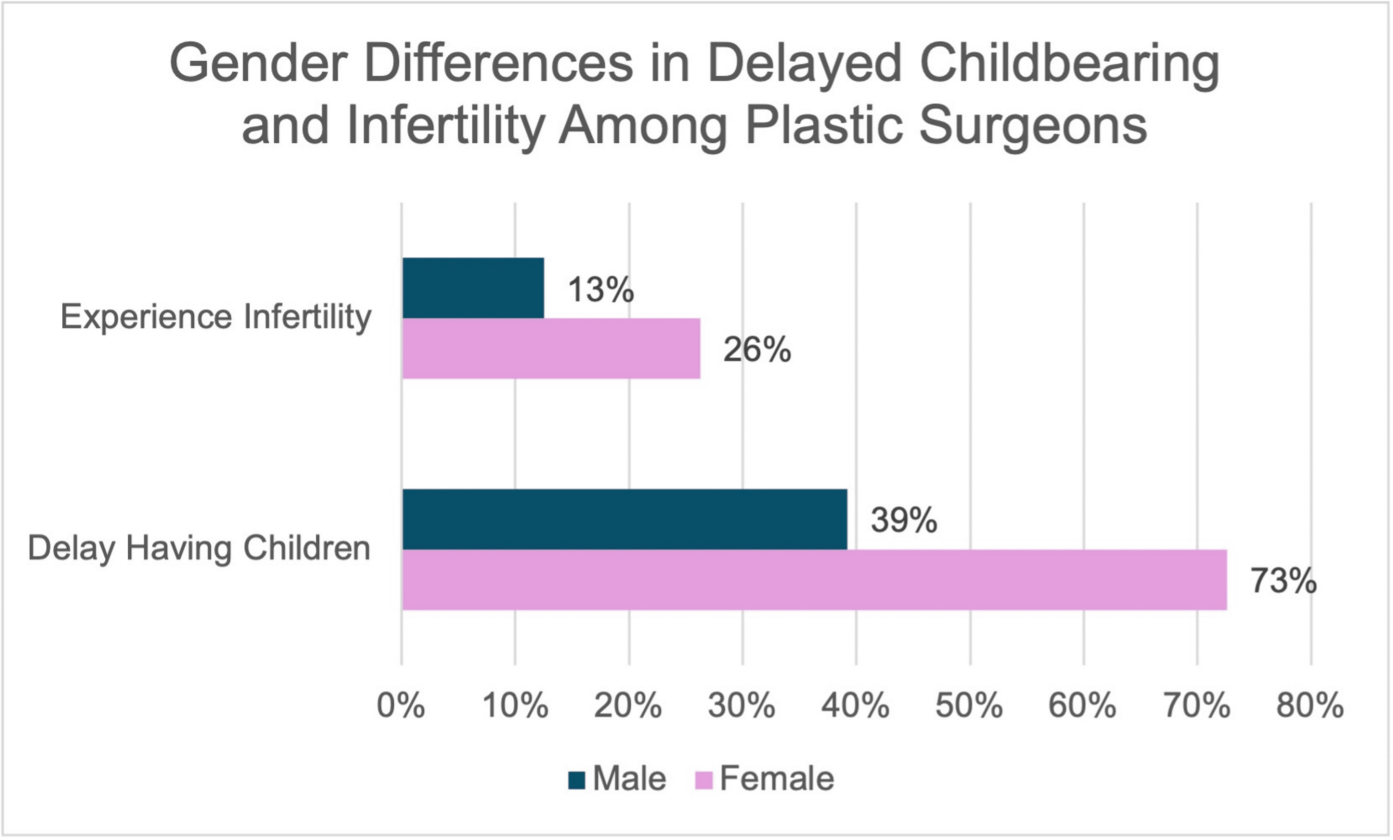
Gender differences in delayed childbearing and infertility among plastic surgeons Adapted from [8]

Several studies show that the most prominent barrier found for women during plastic surgery training is pregnancy. In a survey study of 757 respondents, 309 being female (40.8%), women were nearly twice as likely as men to delay having children (73% female vs. 39% male) and to experience infertility because of the demands of training (26% female vs. 12% male) (Figure 2) [8]. One review reported that female plastic surgeons are more likely to not have children compared with their male colleagues (33%-43% female vs. 2%-12% male, respectively) [9]. Furthermore, the majority of women said there was no discussion about reducing clinical hours before labor, and almost 80% of women who were pregnant during training reported that they worked until the initiation of labor [8]. Another study showed that 76.2% of women felt that their gender was sometimes a disadvantage in career advancement, even though 73.8% of women felt that patients chose them because of their gender [10]. 

An identified barrier that persists throughout residency training is the gender disparity in research publications of trainees at graduation. Female trainees complete their plastic surgery training with fewer publications than their male counterparts (8.89 ± 0.97 female vs. 12.46 ± 1.08 male, p = 0.0394). Male gender was a significant predictor of increased total training publications (p = 0.029) and middle-author publications (p = 0.023) [11]. When an author change occurred in the first-author position, 68% of females were replaced by males, compared to 29% of males being replaced by females. When an author change occurred in the senior author position, 88% of female senior authors were replaced by males, while 19% of male senior authors were replaced by females. Female presenters and abstract senior authors were significantly more likely to be replaced by male first and last authors, respectively (p < 0.001) [7]. Despite these barriers, female representation has increased from 21.84% to 37.31% over the last decade in independent and integrated plastic surgery residency training programs [12].

Barriers Identified After Plastic Surgery Training

Studies highlight that the most significant barriers after plastic surgery training are gender discrepancies in department chair and program director positions and industry payments. 

**Figure 3:**
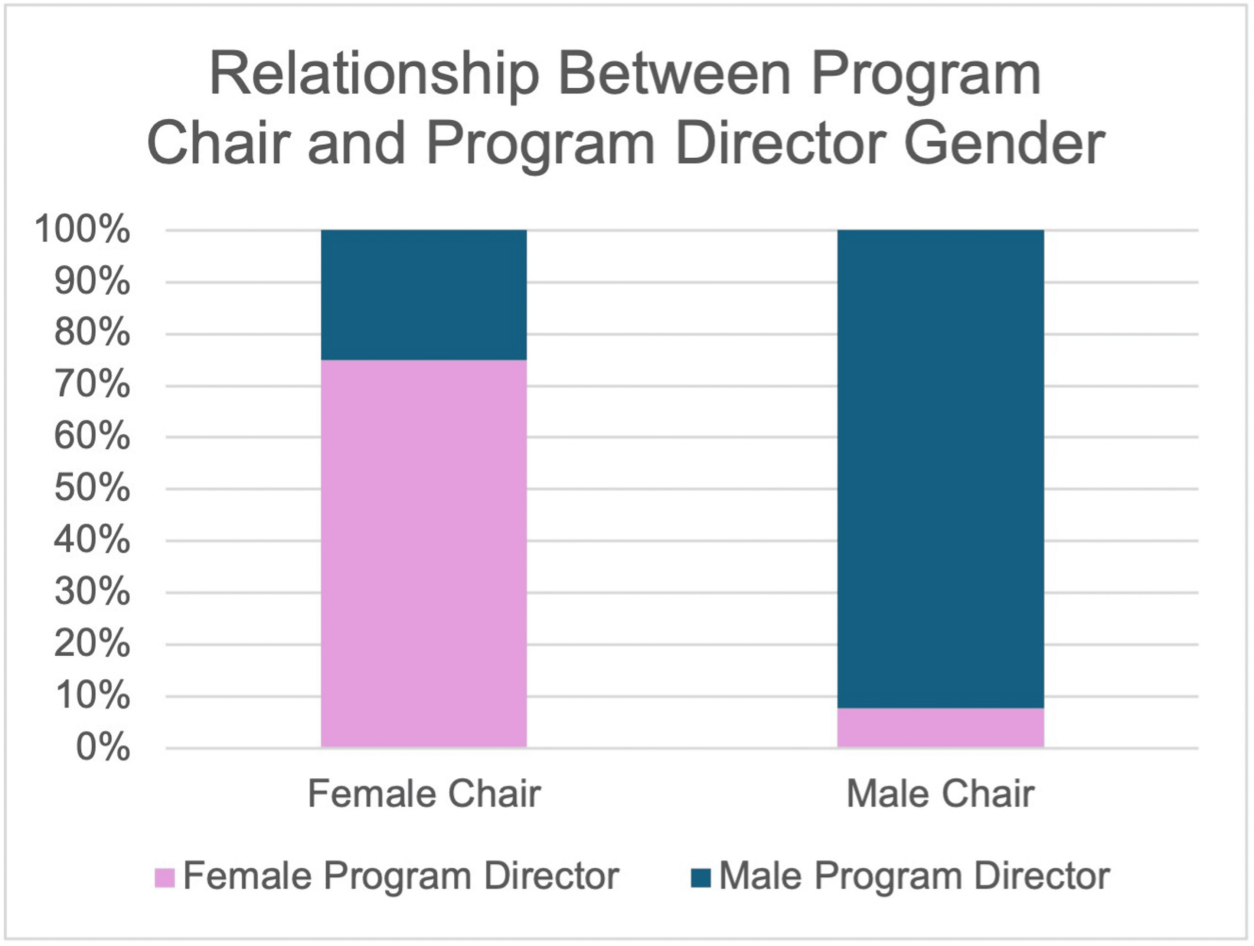
Relationship between program chair and program director gender Adapted from [15]

**Figure 4:**
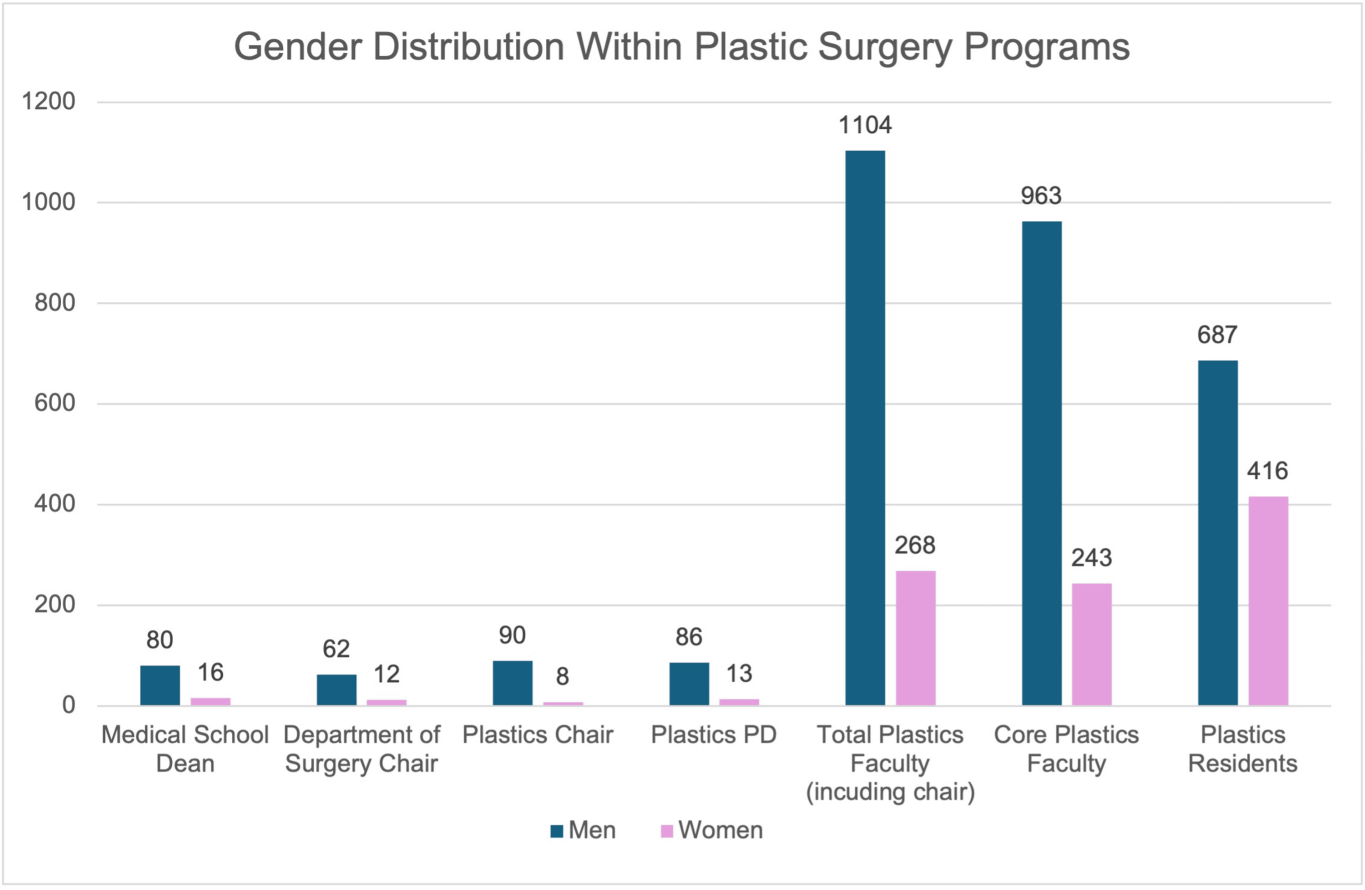
Gender distribution within plastic surgery programs Adapted from [15]

A subgroup analysis of 818 academic plastic surgeons showed a significant gender gap among those holding positions as department chairs or division chiefs (16% male vs. 6% female; p = 0.0037) [13]. Of 99 integrated and independent plastic surgery residency programs, 9 had female department chairs (9.2%) and 13 had female program directors (13.1%). There was no significant difference in the number of years in practice between female and male department chairs (21.3 years female vs. 21.6 years male; p = 0.93). However, female chairs had a significantly higher number of publications, almost double that of their male counterparts (128 female vs. 71.9 male; p < 0.05) [14]. Plastic surgery department chair gender was significantly associated with program director gender (p = 4.0 x 10^-5^), and a female chair was associated with a 45% relative increase in female plastic surgery residents. Out of the programs with a female department chair, 75% had female program directors, while only 7.8% of programs with male department chairs had female program directors (Figure 3). The gender distribution of plastic surgery chair, program director, faculty, and residents in 99 plastic surgery residency programs is shown in Figure 4, with some exclusions due to incomplete data (three programs for medical school dean and department of surgery chair (n = 96), one program for plastic surgery chair (n = 98), and eight programs for residents (n = 91)) [15]. Another study found that out of 1,441 plastic surgery faculty, only 18.3% were women. In addition, female plastic surgeons represented only 26.3% of all assistant professors (p < 0.001), 18.75% of total associate professors (p < 0.001), and 7.8% of full professors (p < 0.001) [16].

Multiple studies investigated the gender disparity in industry payments using the Center for Medicare and Medicaid Services open payment databases. One study found that between 2013 and 2017, $44.4 million in total payments were made from the industry, with $3.35 million made to female plastic surgeons (p < 0.01). Female plastic surgeons were found to receive lower overall payments than male plastic surgeons (median, $3,500 (interquartile range, $800 to $9,500) vs. $4,160.60 (interquartile range, $1,000 to $19,728.20); p < 0.01) [17]. Another study examining industry payments to plastic surgeons in 2017 found that although a similar proportion of male (89.4%) and female (89.8%) academic plastic surgeons received contributions from the industry in 2017, collectively, men received 92% of total funds disbursed. Six-figure payments to a single individual went exclusively to men, with the highest-paid male academic plastic surgeon receiving US $428,429 and the highest-paid female academic plastic surgeon receiving US $82,795 [18]. In a cross-sectional analysis of industry payments to plastic surgeons in 2018, the monetary value of industry contributions was distributed equitably between men and women for all payment types (food and beverage, royalties, consulting fees, speaker fees, and payments for educational purposes), except for speaker fees. When all funded speaker events were analyzed, women received lower median dollar amounts than their male counterparts ($3,675 vs. $7,134, p < 0.0001) [19]. 

Discussion

This narrative literature review has several limitations. This, being a narrative review, we could not conduct a proper risk of bias assessment for each source. Without this evaluation, we could not determine the extent to which biases in individual studies may have influenced our findings. Second, our review compiled information from a range of studies, but we could not conduct a formal certainty of evidence evaluation, which is typical for systematic reviews. This makes it difficult to objectively assess how much confidence can be placed in the findings derived from the included studies. In addition, the intersectionality of other factors, such as race, ethnicity, and socioeconomic status, further complicates barriers that women face in plastic surgery. 

Plastic surgeons require at least 10 years of post-undergraduate education and training. Choosing plastic surgery as a career is likely a decision made early in a student’s career. From the female perspective at the beginning of medical school, the idea of dedicating 10 years of her life to rigorous studies and surgery training implies that she will have little time for personal or social pursuits. For young women considering a career in plastic surgery, making a long-term commitment to pursue this path can be daunting, especially considering the imbalance of female representation at the practicing plastic surgeon level. As a result, many female medical students may assume that plastic surgery is not the field for them. 

A female plastic surgeon can model a work-life balance for a female medical student. In addition, mentorship can help instill a sense of confidence in female medical students interested in pursuing plastic surgery. This is crucial, as confidence often translates into the grit and resilience required to become a plastic surgeon. Without proper mentorship early on, the field of plastic surgery may lose potentially brilliant female surgeons before their training.

Women and men must go through the same training requirements and exams, yet they differ drastically in their reproductive lives. The years of surgery training often coincide with when individuals wish to progress in their personal lives (i.e., marriage, starting a family). Many women feel that becoming pregnant during residency is a burden to their colleagues, even though most women worked until the initiation of labor and did not have a reduction in hours before delivery. The Accreditation Council for Graduate Medical Education (ACGME) and the American Board of Plastic Surgery (ABPS) offer only a four-week maximum for maternity leave during medical training, with the option for an additional two weeks of maternity leave that is “borrowed” from another training year’s vacation time to maintain the mandated 48 clinical weeks per year residency training requirement [11]. This leaves a short amount of time for new mothers to bond with their newborn baby and to heal physically and emotionally, regardless of a difficult birth. Returning to work so quickly can be taxing mentally and physically. This, combined with plastic surgery training, which is demanding on its own, could persuade women that plastic surgery as a profession is not for them. On the other hand, women who choose to pursue plastic surgery may make peace with having to delay childbearing until after training. Items to be examined to improve the gender disparity include practices surrounding pregnancy in plastic surgery residency, maternity leave, breastfeeding policies, and delays in starting a family. 

Research is important at all stages of medical/surgical training and is especially crucial for applicants applying to integrated plastic surgery programs. Many presenters are at the early stages of their careers (medical students or residents), and the imbalance between female and male presenters at major plastic surgery conferences mirrors the gender discrepancy seen in the current plastic surgeon demographics. This suggests that achieving equal representation at conferences could contribute to equal representation at the plastic surgeon level. This begins by encouraging women to present at conferences at every stage and making these opportunities more accessible to young women interested in pursuing plastic surgery as a career. Furthermore, having mentors (both male and female), who recognize the potential in young women pursuing plastic surgery, can contribute to equalizing the availability of research opportunities across genders. 

In the context of career advancement, the data demonstrated that female plastic surgery chairs had twice as many publications as their male counterparts in the same positions. This suggests that women must do more to prove their competency and hold the same space. Furthermore, when female senior authors were replaced, the overwhelming majority were replaced by men. This could suggest that women do not have a similar abundance of networking opportunities as their male counterparts. From a social perspective, women, more than men, may be seen as aggressive or over the top if they are assertive in building connections, making change, or being involved in social activities after professional engagements. Women may perceive that they must work harder to create opportunities that their male counterparts might have an easier time obtaining, which can further discourage women from entering or staying in the field. 

Regarding studies showing that women receive fewer industry payments than their male counterparts, it is difficult to conclude that this discrepancy is solely due to gender disparity in plastic surgery. Across various industry payment categories (food and beverage, royalties, consulting fees, speaker fees, and payments for educational purposes), compensation was distributed equitably between men and women, except for speaker fees. Women received significantly lower median dollar amounts than their male counterparts in the context of funded speaker events. Potential causes for this discrepancy in speaker fees require further studies.

While there are a multitude of barriers that exist for women in plastic surgery, the percentage of female plastic surgery residents in the United States has steadily increased over the last decade. Because women make up almost 40% of the current plastic surgery resident population [12], it is warranted now more than ever to reexamine ways in which equity can be achieved for women pursuing a career in plastic surgery.

## Conclusions

Increasing the fraction of female surgeons in plastic surgery is important to improve diversity within the field. Despite the growing proportion of women entering medical school training and a similar proportion of female and male applicants applying to integrated plastic surgery residency, there still exists a large gender gap between women and men practicing plastic surgery. Evidence of barriers affecting women in plastic surgery is shown by the gender disparity seen in research presenters, the rates of delayed childbearing and infertility, the number of publications plastic surgery trainees graduate with, the proportion of female program directors and department chairs, and industry payments in the form of speaker fees. Ways to combat some of these barriers include providing more opportunities for female medical students to connect with female plastic surgery mentors, allowing more residents to modify maternity leave (and paternity leave) in a way that supports families, potentially building in time for research during leave, and creating more flexible program policies to allow additional time off in the case of a complicated birth. More studies are required to examine in detail the various barriers women face in plastic surgery at every stage of their careers and to potentially implement systemic changes that enhance equality for women pursuing a career in plastic surgery. 
